# Age at Menarche and Risks of All-Cause and Cardiovascular Death: A Systematic Review and Meta-Analysis

**DOI:** 10.1093/aje/kwu113

**Published:** 2014-06-11

**Authors:** Dimitrios Charalampopoulos, Andrew McLoughlin, Cathy E. Elks, Ken K. Ong

**Keywords:** cardiovascular disease, death rate, menarche, mortality, puberty

## Abstract

We conducted a systematic review and meta-analysis to investigate the associations between menarcheal age and all-cause and cardiovascular death. Medline, Embase, Scopus, and Web of Knowledge were searched for articles published prior to March 2013 reporting on the associations between menarcheal age and death from all causes or from cardiovascular disease (total cardiovascular disease, ischemic heart disease (IHD), and stroke) in adult women. Nine articles were eligible for inclusion; these reported 5 estimates each for death from all causes and total cardiovascular death, 6 estimates for IHD, and 7 estimates for death from stroke. Our meta-analysis showed that each 1-year increase in age at menarche was associated with a 3% lower relative risk of death from all causes (pooled hazard ratio = 0.97, 95% confidence interval: 0.96, 0.98) with low heterogeneity (*I*^2^ = 32.2%). Meta-analysis of 2 cohorts showed a higher risk of death from all causes for women who experienced early menarche (at <12 years of age) versus “not early” menarche (at ≥12 years of age) (pooled hazard ratio = 1.23, 95% confidence interval: 1.10, 1.38; *I*^2^ = 0%). An inverse association between age at menarche and death from IHD was observed only among nonsmoking populations or populations with low prevalence of smoking. We found no evidence of association between age at menarche and death from all cardiovascular diseases or stroke. Early menarche was consistently associated with higher risk of death from all causes. Further studies are needed to clarify the role of menarcheal age on cardiovascular outcomes and to investigate the potential modifying role of smoking.

Menarche, defined as the first menstrual period in a woman's life, is a landmark of pubertal development in girls. Although menarche occurs quite late in the chain of pubertal events, normally following breast development and peak height velocity, it is regarded as a milestone in a woman's life, signifying the onset of her reproductive capacity (1). Age at menarche has received a great deal of attention over the last few years as having important health implications, either direct or indirect.

There is a growing body of epidemiologic studies suggesting an association between early menarche and risk factors for cardiovascular disease and metabolic syndrome in both adolescent girls and adult women (2–5). Results from a recent meta-analysis of 10 cohort studies showed that early menarche (at <12 years of age) was associated with higher adult body mass index (BMI) (weight (kg)/height (m)^2^), with a mean BMI difference of 0.34 between women who experienced early menarche versus those who experienced menarche at 12 or more years of age (6). Moreover, there is evidence for a connection between menarche and morbidity from cardiovascular disease (CVD), with results from meta-analysis suggesting an association between early menarche and higher risk of CVD-related events (risk ratio = 1.15, 95% confidence interval (CI): 1.02, 1.28) (6). Also, early menarche has been associated with a higher risk of breast cancer (7), whereas there is some evidence that it might have a protective effect against death from hip fracture (8).

Although a link between the timing of menarche and cardiovascular morbidity has been shown (6), there was insufficient evidence in that earlier review to draw conclusions on the relevance of menarcheal age on other important health outcomes, including death from all causes and from cardiovascular diseases. To the best of our knowledge, no focused systematic review and meta-analysis has yet been conducted on this topic. Therefore, the aim of the current study was to systematically review and synthesize existing evidence on the association between menarcheal age and the risks of death from all causes and from cardiovascular diseases in women.

## METHODS

### Search strategy and study selection

We systematically searched the Medline (from its inception), Embase (from 1974), Scopus (from its inception), and Web of Knowledge (from its inception) databases through March 2013 for relevant citations. We used a combination of keywords and medical subject heading (MeSH) terms to generate 3 subsets of citations: 1 relating to exposure, 1 on indexing outcomes, and 1 for study designs. Results were then combined with “AND” and limited to humans. The search was not limited by language of publication. A full list of the search terms we used is included in Web Appendix 1 available at http://aje.oxfordjournals.org/.

Eligible studies examined the association between menarcheal age and death from all causes and/or from cardiovascular diseases (including death from total CVD, ischemic heart disease (IHD), or stroke) in humans and provided an appropriate effect estimate for this association (i.e., odds ratio, risk ratio, rate ratio, or hazard ratio). Although it was expected that cohort studies would offer the most appropriate design to test the association between menarcheal age and the risk of death, we included studies of any observational design (e.g., cohort, cross-sectional, and case-control). We excluded studies if the outcome of interest included nonfatal cardiovascular events or if only women with specific health conditions were selected (e.g., women with breast cancer). Also, letters, editorials, reviews, notes, and studies conducted in animals were excluded.

Retrieved citations were entered into a reference management library (EndNote, Thomson-Reuters Corp., New York, New York), and duplicates were removed automatically and by hand. Titles and abstracts of unique citations were initially screened for meeting eligibility criteria. Full texts of potentially eligible articles were retrieved. Titles, abstracts, and full texts were reviewed independently by 2 reviewers (D.C. and A.M.), and disagreements were resolved through open discussion. Bibliographies of the selected articles were hand-searched for additional eligible studies, and all corresponding authors of the selected articles were contacted for any known published or unpublished relevant studies. We also searched the Embase, Web of Knowledge, and Scopus databases for conference proceedings.

### Quality evaluation

Quality assessment of the included studies was based on the Newcastle-Ottawa Scale for cohort studies (9) (Web Appendix 2). The scale consists of 8 items, of which 7 were applicable to our study question. Items are grouped into 3 domains (i.e., selection, comparability, and outcome). Two independent reviewers (D.C. and A.M.) read and scored each of the studies, giving a maximum of 8 stars to any individual study. We assigned total scores of 0–3, 4–5, and 6–8 stars for low-, moderate-, and high-quality studies, respectively. Any discrepancies between the reviewers were resolved with a joint reassessment, after which a consensus was reached.

### Data extraction

From each study, we extracted a predetermined set of data, including name of the first author, year of publication, sample characteristics, study settings, follow-up details, number of deaths, effect measures, and confounders. Adjusted hazard ratios were used as the main measure of association across studies. For the association between menarcheal age and mortality outcomes, the following effect estimates were entered into the meta-analysis, if available: 1) the hazard ratio associated with a 1-category increase in menarcheal age group (linear effect); 2) the hazard ratio for the comparison between early menarche (at <12 years of age) and “not early” menarche (at ≥12 years of age); and 3) the hazard ratio comparing the earliest versus the median menarcheal age group. The hazard ratio for a 1-category increase in menarcheal age was assumed to approximate the hazard ratio for a 1-year increase in age at menarche, given the large number of menarcheal age groups used in most studies. Menarcheal age of less than 12 years was chosen to define early menarche because this age has been conventionally used by most studies. When studies reported hazard ratios from models with different levels of adjustment, the most comprehensively adjusted hazard ratio was selected. Stratified results were combined within each study before using them in the meta-analysis.

### Data analysis

We analyzed data both descriptively and through meta-analysis. Estimates were pooled by using a random-effects model because differences in populations and settings between studies could not easily justify a common effect size. A separate analysis using a fixed-effect model was also conducted and, unless otherwise stated, no differences in the summary estimates were found. We calculated the *I*^2^ statistic to quantify the magnitude of between-study heterogeneity, and we considered values of 50% or less, 51%–75%, and 76% or more to be indicative of low, moderate, and high heterogeneity, respectively (10). We explored whether heterogeneity could be reduced by removing studies 1 at a time from the meta-analysis. Moreover, we investigated potential sources of heterogeneity by conducting subgroup analyses. Results are presented as pooled hazard ratios and their 95% confidence intervals. We generated forest plots sorted by level of precision to visually assess hazard ratios across studies. Finally, funnel plots were generated to evaluate the possibility of publication bias. All analyses were performed using Stata, version 10.0, software (StataCorp LP, College Station, Texas).

## RESULTS

**Figure 1.**
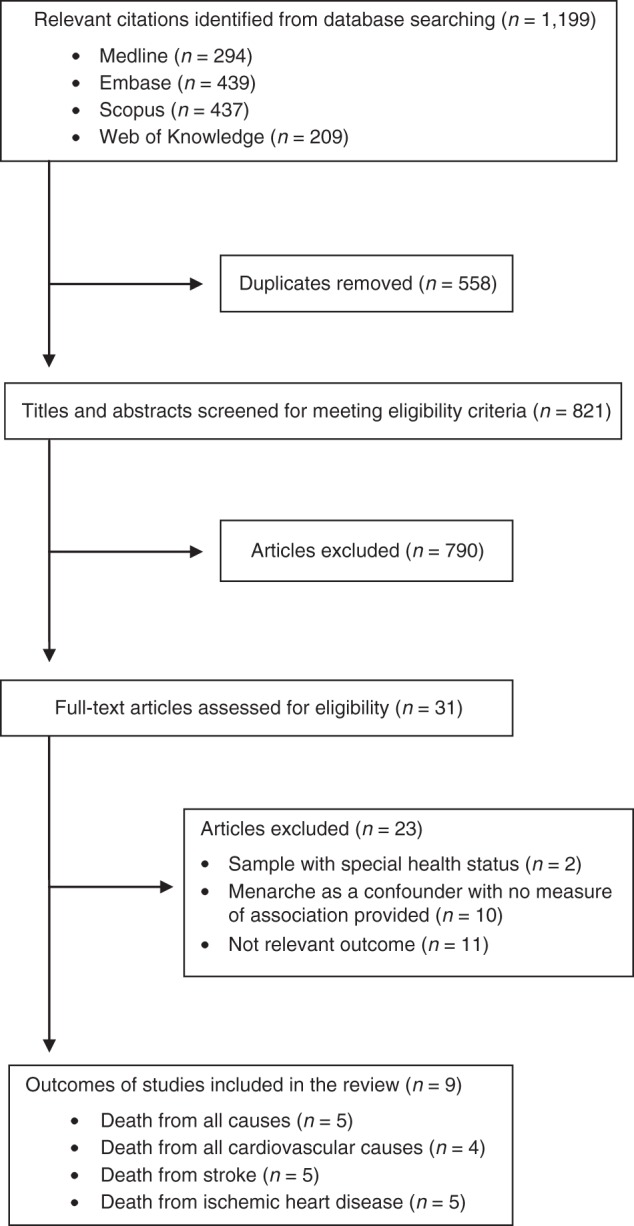
Flow diagram showing the search strategy used in the current review.

Through electronic searches, we identified 821 unique articles, all of which were published in English. No additional studies were identified through manual searches or contact with authors. Of the 821 articles, 790 were excluded on the basis of titles and abstracts, leaving 31 articles for further evaluation. After screening full texts of these articles for meeting eligibility criteria, we included 9 articles in the current review (Figure 1).

### Study characteristics

The 9 included articles provided data from 8 independent cohorts and, although they were published between 2006 and 2012, they covered a study period of 50 years (1959–2009). Sample sizes ranged from 1,031 to 267,400 women. In all studies, menarcheal age was self-reported and grouped into 5 or more categories, with the exception of 3 studies (11–13). Ascertainment of mortality outcomes was based on record linkage with official death certificates, and *International Classification of Diseases* codes were used to identify deaths from cardiovascular causes. All studies used Cox proportional hazards regression models for their analyses. Of the 9 eligible articles, 3 articles (12, 14, 15) included death from all causes as their only outcome, 4 studies (11, 13, 16, 17) included only death from cardiovascular causes, and 2 studies (18, 19) reported on both outcomes (Tables 1 and 2).

**Table 1.** Characteristics of Studies Included in the Review for the Association Between Menarcheal Age and Death From All Causes

First Author, Year (Reference No.)	Location	Study Period	Study Name/Source	No. of Participants	Mean Years of Follow-Up	Total Person-Years	No. of Deaths	Mean Age at Baseline, years	Age Range, years	Median MA, years	No. of MA Groups
Jacobsen, 2007 (14)	Norway	1959–1997	Norwegian Study	61,319	28.4	1,740,931	36,114	49.4	32–74	14	6
Tamakoshi, 2011 (15)	Japan	1988–2006	JACC Study	55,128	14.5	818,379	6,967	57.1	40–79	14	7
Lakshman, 2009 (19)	United Kingdom	1993–2008	EPIC-Norfolk	15,807	12^a^	185,220	1,903	58.6	40–79	13	5
Jacobsen, 2009 (18)	United States (California)	1976–1988	Adventist Health Study	19,462	11.1	215,486	3,313	55.1	26–101	13	6
Giles, 2010 (12)	Australia	1992–2007	ALSA	1,031	7.3	7,526	673	77.3	65–103	14	2

Abbreviations: ALSA, Australian Longitudinal Study of Ageing; EPIC, European Prospective Investigation of Cancer; JACC, Japan Collaborative Cohort Study for Evaluation of Cancer Risk; MA, menarcheal age.

^a^ Value expressed as median.

**Table 2.** Characteristics of Studies Included in the Review for the Association Between Menarcheal Age and Cardiovascular Death

First Author, Year (Reference No.)	Location	Study Period	Study Name/Source	No. of Participants	Mean Years of Follow-Up	Total Person-Years	Mean Age at Baseline, years	Median MA, years	No. of MA Groups
Gallagher, 2011 (17)	China (Shanghai)	1989–2000	Nonsmoking Textile Workers Study	267,400	9.6	2,565,433	43^a^	15	5
Cui, 2006 (16)	Japan	1988–1999	JACC Study	37,965	10	379,094	40–79^b^	15	5
Lakshman, 2009 (19)	United Kingdom	1993–2007	EPIC-Norfolk	15,807	10.6^a^	159,199	58.6	13	5
Jacobsen, 2009 (18)	United States (California)	1976–1988	Adventist Health Study	19,462	11.1	215,485	55.1	13	6
Mueller, 2012 (13)	Singapore	1993–2009	SCHS	34,022	13.5	460,374	56.3	13.5	4
Chang, 2011 (11)	Korea	1985–2005	KCS	3,257	17.6	48,313	66.8	17.6	3

Abbreviations: EPIC, European Prospective Investigation of Cancer; JACC, Japan Collaborative Cohort Study for Evaluation of Cancer Risk; KCS, Kangwha Cohort Study; MA, menarcheal age; SCHS, Singapore Chinese Health Study.

^a^ Value expressed as median.

^b^ Values expressed as range.

### Quality assessment

Table 3 presents results of the quality assessment under the Newcastle-Ottawa Scale. All of the included studies were of adequate quality. Five studies received between 4 and 5 stars, indicating moderate quality, and 4 studies received 7 stars, indicating high quality. No low-quality studies were identified.

**Table 3.** Quality Evaluation of all Studies Included in the Review Based on the Newcastle-Ottawa Scale for Quality Assessment of Cohort Studies^a^

First Author, Year (Reference No.)	Selection^b^						Comparability		Outcome						Total No. of Stars	Quality Level
	Representativeness of Exposed Cohort		Selection of Nonexposed Cohort		Exposure Ascertainment		Level of Adjustment (Analysis/Design)		Outcome Assessment		Long Enough Follow-Up		Adequate Follow-Up			
	Answer	No. of Stars	Answer	No. of Stars	Answer	No. of Stars	Answer	No. of Stars	Answer	No. of Stars	Answer	No. of Stars	Answer	No. of Stars		
Mueller, 2012 (13)	C	0	A	1	B	1	A	2	B	1	A	1	B	1	7	High
Chang, 2011 (11)	C	0	A	1	B	1	A	2	B	1	A	1	B	1	7	High
Jacobsen, 2007 (14)	B	1	A	1	B	1	A	1	B	1	A	1	D	0	6	High
Tamakoshi, 2011 (15)	B	1	A	1	C	0	A	1	B	1	A	1	D	0	5	Moderate
Lakshman, 2009 (19)	B	1	A	1	C	0	A	2	B	1	A	1	D	0	6	High
Jacobsen, 2009 (18)	C	0	A	1	C	0	A	1	B	1	A	1	D	0	4	Moderate
Giles, 2010 (12)	C	0	A	1	C	0	A	2	B	1	A	1	D	0	5	Moderate
Cui, 2006 (16)	C	0	A	1	C	0	A	2	B	1	A	1	D	0	5	Moderate
Gallagher, 2011 (17)	C	0	A	1	C	0	A	1	B	1	A	1	D	0	4	Moderate

^a^ A study can be awarded a maximum of 1 star for each item within the selection and outcome categories and a maximum of 2 stars for comparability (1 star was given for studies adjusting for age and 2 stars for studies also controlling for at least 1 indicator of socioeconomic status, for example, education, income, and at least 1 of the following lifestyle factors: physical activity, alcohol, or diet). Stars are awarded on the basis of answers provided for each item (A, B, C, or D). The scale ranges from 0 to 8 stars. We assigned scores of 0–3, 4–5, and 6–8 for low-, moderate-, and high-quality studies, respectively. For a detailed view of the scale, see Web Appendix 2.

^b^ The fourth item of the selection category (outcome not present at the time of enrollment) was omitted because it was not applicable to the current studies.

Recall bias in menarcheal age ascertainment was the most common source of bias. The effect of recall bias is likely to be lower in studies that used structured interviews (11, 13, 14) for data elicitation and higher in studies with subjects of older age because of poor memory. Most studies adjusted for potentially important confounders with the exception of 3 studies, in which key lifestyle (i.e., diet, exercise) (17, 18) and socioeconomic factors (15, 17, 18) were not considered. In terms of sample recruitment, 3 cohorts selected population-based samples that were representative of the target population (14, 15, 19). Two cohorts recruited samples with special health behaviors (e.g., women socialized in the Adventist Church (18) and nonsmoking textile workers (17)). Three studies (11, 14, 16) used restricted samples of older postmenopausal women, hence limiting their generalizability. Follow-up of participants was adequately long in all studies; however, only 2 studies (11, 13) provided information for noninformative censoring related to losses to follow-up. Visual inspection of the funnel plot showed no obvious asymmetry indicating publication bias for cardiovascular death outcomes (Web Figure 1).

### Death from all causes

Five cohort studies (12, 14, 15, 18, 19) investigated the association between menarcheal age and death from all causes (Table 4). In total, 152,747 women were followed for a mean period of 7.3–28.4 years, providing 2,967,542 person-years of follow-up. Two cohorts (14, 19) were from Europe, and 1 each was from Japan (15), Australia (12) and the United States (18). Of the 5 studies, 4 studies (14, 15, 18, 19) reported significant inverse associations between menarcheal age and the risk of death from all causes.

**Table 4.** Measures of Association and Adjustments in Studies Examining the Association Between Menarcheal Age and the Risk of Death From All Causes

First Author, Year (Reference No.)	Early vs. “Not Early” MA^a^		1-Category Increase in MA Group		Earliest vs. Median MA Group		Covariates in Fully Adjusted Model
	HR	95% CI	HR	95% CI	HR	95% CI	
Jacobsen, 2007 (14)			0.98	0.97, 0.98	1.09	1.05, 1.13	Age, birth cohort^b^
Tamakoshi, 2011 (15)			0.97	0.95, 0.99^c^	1.16	1.01, 1.32	Age, location, smoking, alcohol intake, exercise, sleeping, parity, menopausal status, and BMI^d^
Lakshman, 2009 (19)	1.22	1.07, 1.39	0.96	0.93, 0.99	1.16	1.01, 1.34	Age, smoking, alcohol intake, exercise, education, parity, occupational social class, BMI, oral contraceptive use, hormone replacement therapy use, and waist circumference
Jacobsen, 2009 (18)			0.955	0.93, 0.98	1.45	1.18, 1.78	Age
Giles, 2010 (12)	1.28	0.99, 1.65	0.76	0.56, 1.04	1.28	0.99, 1.65	Age, location, smoking, exercise, BMI, parity, age at menopause, number of reproductive years, and health variables (self-rated health, cognitive function, and number of morbid conditions)

Abbreviations: BMI, body mass index; CI, confidence interval; HR, hazard ratio; MA, menarcheal age.

^a^ Early menarcheal age is defined as less than 12 years; “not early” menarcheal age is defined as 12 years or older.

^b^ Results were not substantially affected after adjustment for occupation, parity, residence, marital status, age at first birth, and BMI, but no further details were provided.

^c^ The hazard ratio comes from a model with a significant linear effect (*P* = 0.004), in which women with extreme menarcheal ages of 9 years (*n* = 9) and 18–20 years (*n* = 5,156) were excluded.

^d^ Weight (kg)/height (m)^2^.

In all of the 5 studies of death from all causes, the linear estimates approximate trends per 1-year increase in age at menarche. Four studies grouped together the highest or lowest ages at menarche into single categories, but the intermediate categories were always separated by 1 year (Web Table 1). By meta-analysis, each 1-year increase in age at menarche was associated with a 3% lower relative risk for death from all causes (pooled hazard ratio (HR) = 0.97, 95% CI: 0.96, 0.98) with low heterogeneity between studies (*I*^2^ = 32.2%). Three of the reported estimates (12, 15, 19) were from models that included adjustment for adult BMI, which is a potential mediator between age at menarche and mortality outcomes. A meta-analysis of this subgroup showed that age at menarche remained significantly inversely associated with death from all causes, with little heterogeneity between the estimates (HR = 0.97, 95% CI: 0.95, 0.98; *I*^2^ = 23.5%).

**Figure 2.**
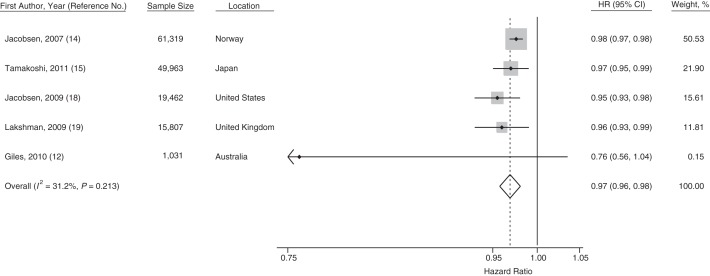
Forest plot displaying a random-effects meta-analysis of the adjusted hazard ratios (HRs) for death from all causes associated with a 1-category (usually corresponding to a 1-year) increase in menarcheal age. Boxes represent the hazard ratios for each individual study with the size of the box reflecting the weight assigned to the study. Dotted vertical line represents the combined estimate. The width of the diamond illustrates the 95% confidence intervals (CIs) around the combined estimate.

**Figure 3.**
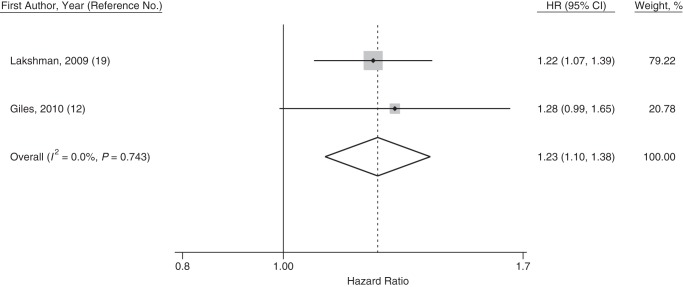
Forest plot displaying a random-effects meta-analysis of the adjusted hazard ratios (HRs) of death from all causes for the comparison between women with early menarche (at <12 years of age) and “not early” menarche (at ≥12 years of age). Boxes represent the hazard ratios for each individual study with the size of the box reflecting the weight assigned to the study. Dotted vertical line represents the combined estimate. The width of the diamond illustrates the 95% confidence intervals (CIs) around the combined estimate.

**Figure 4.**
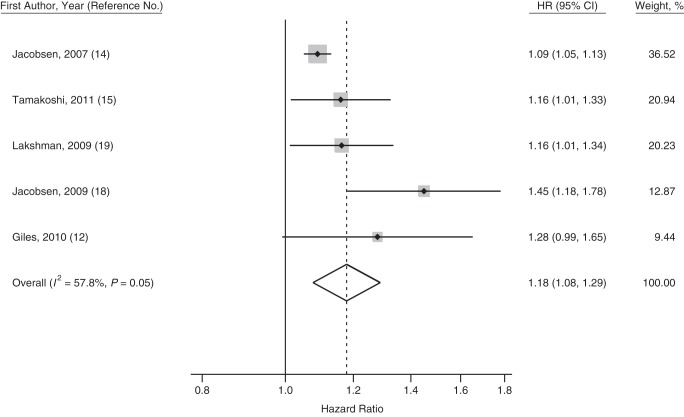
Forest plot displaying a random-effects meta-analysis of the adjusted hazard ratios (HRs) of death from all causes comparing women in the earliest versus the median menarcheal age group. Boxes represent the hazard ratios for each individual study with the size of the box reflecting the weight assigned to the study. Dotted vertical line represents the combined estimate. The width of the diamond illustrates the 95% confidence intervals (CIs) around the combined estimate.

Meta-analysis of the estimates comparing the earliest versus the median menarcheal age group showed that women in the earliest menarcheal age group had a higher risk of death from all causes compared with those in the median menarcheal age group (pooled HR = 1.18, 95% CI: 1.08, 1.29; *I*^2^ = 57.8%). Two studies (12, 19) also reported estimates for early menarche using the same definition (<12 years of age vs. ≥12 years of age); these resulted in a pooled hazard ratio of 1.23 (95% CI: 1.10, 1.38, *I*^2^ = 0%) for the risk of death associated with early menarche. Forest plots are presented in Figures 2–4.

**Figure 5.**
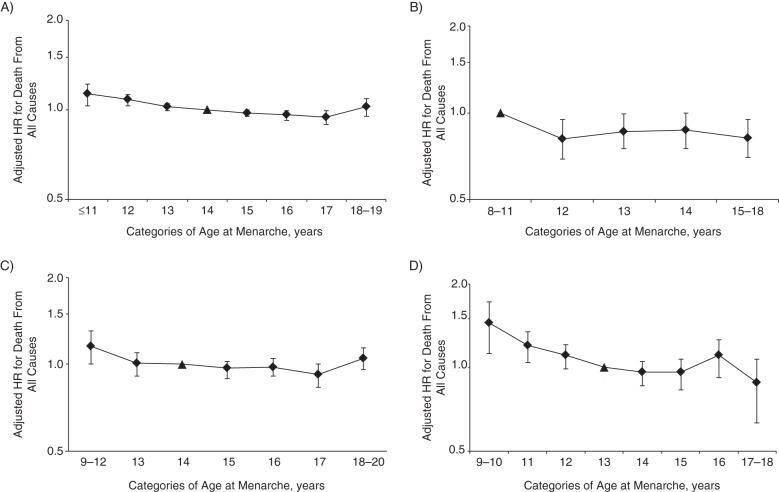
Adjusted hazard ratios (HRs) (on a log scale) of death from all causes across categories of age at menarche in completed whole years from studies by A) Jacobsen et al. (14), B) Lakshman et al. (19), C) Tamakoshi et al. (15), and D) Jacobsen et al. (18). Triangles depict reference categories. Bars, 95% confidence intervals.

Four of 5 articles (14, 15, 18, 19) reported hazard ratios for death from all causes by separate menarcheal age categories, allowing assessment of the shape of the association (Figures 5A–D). The relationship between age at menarche and death from all causes appeared to be generally linear. Jacobsen et al. (14) and Tamakoshi et al. (15) reported slightly higher risks of death from all causes in women with the highest menarcheal ages (i.e., U-shaped associations); however, no studies reported formal tests for nonlinearity.

### Death from cardiovascular causes

**Figure 6.**
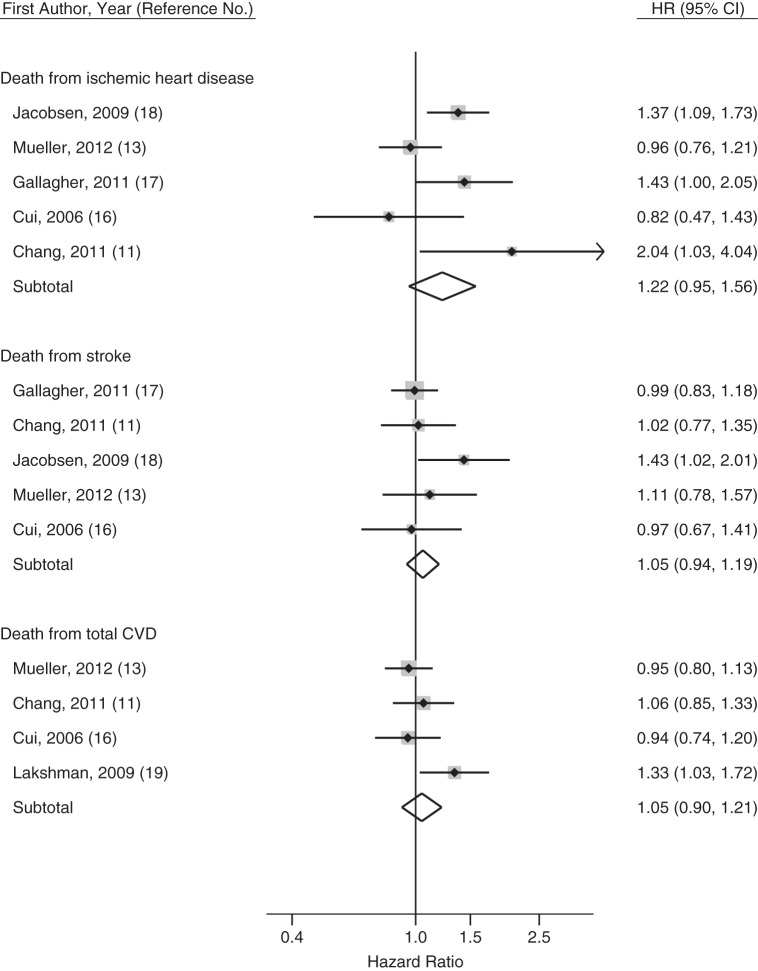
Forest plot of the adjusted hazard ratio (HR) for ischemic heart disease, stroke, and total cardiovascular disease (CVD) death comparing women in the earliest versus the median menarcheal age group. Weights are from a random-effects model. Boxes represent the hazard ratios for each individual study with the size of the box reflecting the weight assigned to the study. Dotted vertical line represents the combined estimate. The width of the large diamonds illustrates the 95% confidence intervals (CIs) around the combined estimate.

Overall, 6 cohorts with a total sample of 377,913 women examined the relationship between menarcheal age and some measure of cardiovascular death, providing 3,827,898 person-years of follow-up (Table 5 and Web Table 2). Four cohorts (11, 13, 16, 17) consisted of Asian subjects, 1 cohort was conducted in the United Kingdom (19), and 1 was conducted in the United States (18). Because only 1 study modeled age at menarche as a linear exposure (18), meta-analysis of cardiovascular death outcomes was based on reported hazard ratios for the earliest versus the median menarcheal age group. Results are presented below and shown in Figure 6.

**Table 5.** Outcome Definitions, Number of Deaths, Measures of Association, and Adjustments in Studies Examining the Association Between Menarcheal Age and Cardiovascular Death Outcomes

First Author, Year (Reference No.)	Cause of Death	ICD Codes	No. of Deaths	Earliest vs. Median MA Group		Covariates in Fully Adjusted Model
				HR	95% CI	
Gallagher, 2011 (17)	IHD	410–414^a^	494	1.44	1.00, 2.05	Age
	IS	434^a^	699	1.05	0.75, 1.45	
	HS	431^a^	1,815	0.97	0.79, 1.19	
Cui, 2006 (16)	IHD	410–414^a^ and I20–I25^b^	178	0.82	0.47, 1.42	Age, smoking, alcohol intake, marital status, education, menopausal status, BMI^c^, history of diabetes, and hypertension
	Stroke	430–438^a^	487	0.97	0.67, 1.41	
	Total CVD	390–459^a^ and I01–I99^b^	1,010	0.94	0.74, 1.20	
Lakshman, 2009 (19)	Total CVD	401–448^a^ and I10–I79^b^	640	1.33	1.03, 1.72	Age, smoking, alcohol intake, exercise, education, parity, occupational social class, oral contraceptive use, and hormone replacement therapy use
Jacobsen, 2009 (18)	IHD	410–414^a^	809	1.37	1.09, 1.73	Age
	Stroke	430–438^a^	378	1.43	1.02, 2.01	
Mueller, 2012 (13)	*Ever Smokers*					
	Total CVD	394–459^a^	341	0.65	0.41, 1.03	Age, year of interview, dialect, education, exercise, diet, alcohol intake, smoking, parity, menopausal status, oral contraceptive use, hormone replacement therapy use, BMI, diabetes, and hypertension
	IHD	410–414.9 and 427.5^a^	194	0.75	0.42, 1.33	
	Stroke	430–438^a^	92	0.68	0.27, 1.75	
	*Never Smokers*					
	Total CVD	394–459^a^	1,511	1.02	0.84, 1.24	
	IHD	410–414.9 and 427.5^a^	804	1.01	0.78, 1.30	
	Stroke	430–438^a^	465	1.2	0.84, 1.70	
Chang, 2011 (11)	Total CVD	I00–I99^b^	478	1.06	0.85, 1.33	Age, education, occupation, alcohol, smoking, BMI, and hypertension
	IHD	I20–I25^b^	47	2.04	1.02, 4.0	
	Stroke	I60–I69^b^	297	1.02	0.78, 1.35	

Abbreviations: BMI, body mass index; CI, confidence interval; CVD, cardiovascular disease; HR, hazard ratio; HS, hemorrhagic stroke; ICD, *International Classification of Diseases*; IHD, ischemic heart disease; IS, ischemic stroke; MA, menarcheal age.

^a^ Codes based on the *International Classification of Diseases, Ninth Revision*.

^b^ Codes based on the *International Classification of Diseases, Tenth Revision*.

^c^ Weight (kg)/height (m)^2^.

#### Total cardiovascular deaths

Four articles (11, 13, 16, 19) reported 5 estimates for total CVD deaths, of which only 1 (13) reported a significant inverse association with age at menarche, but only among never smokers. By meta-analysis, the hazard ratio for total CVD deaths comparing the earliest versus the median menarcheal age group was 1.05 (95% CI: 0.90, 1.21; *I*^2^ = 42.6%). Exclusion of the only European study (19) completely reduced the heterogeneity (pooled HR = 0.98, 95% CI: 0.87, 1.10; *I*^2^ = 0%).

#### Death from ischemic heart disease

Five articles (11, 13, 16–18) reported 6 estimates for death from IHD. Meta-analysis showed that women in the earliest menarcheal age group had a tendency toward higher risk of death from IHD compared with those in the median menarcheal age group (pooled HR = 1.22, 95% CI: 0.95, 1.56). There was moderate heterogeneity between studies (*I*^2^ = 59.1%), which could not be explained by exclusion of any single study. Three studies reported a significant linear trend between earlier age at menarche and higher risk of death from IHD; 1 study (17) included only nonsmoking women, 1 study (18) included a sample with a very low prevalence of smoking (2%), and the third study (13) reported a modifying role of smoking with a significant apparent effect observed only among never smokers (significant interaction). Furthermore, the 2 studies that reported no significant association included only postmenopausal women. A further meta-analysis of the subgroup of 3 studies that included nonsmoking populations or populations with low prevalence of smoking showed an increased risk for the earliest versus the median menarcheal age group with borderline significance (pooled HR = 1.24, 95% CI: 1.00, 1.55) and slightly reduced heterogeneity (*I*^2^ = 47.8%). Meta-analysis of the above 3 estimates by using a fixed-effect model yielded a statistically significant effect (pooled HR = 1.24, 95% CI: 1.06, 1.44).

#### Death from stroke

Five articles (11, 13, 16–18) reported 7 estimates on death from stroke. One article (17) provided results for different types of stroke, whereas 1 study (13) reported separate results for ever smokers and never smokers because of statistically significant interaction. Only 1 study reported a significant linear trend between menarcheal age and risk of death from stroke (17). Meta-analysis of the estimates for risk of death from stroke showed no significant effect for the earliest versus the median menarcheal age group comparison (pooled HR = 1.05, 95% CI: 0.94, 1.19, *I*^2^ = 0%).

## DISCUSSION

Our meta-analysis showed a 3% lower relative risk of death from all causes associated with each 1-year delay in menarche, with consistent findings across studies. There was some suggestion that the association was nonlinear, with a 23% higher relative risk of death in women with early menarche (at <12 years of age), but no obvious protection of late menarche. With regard to cardiovascular-specific death, some evidence for an inverse association between menarcheal age and death from IHD was found only among nonsmoking women or those with low prevalence of smoking, in whom the relative risk of death from IHD was 24% higher in the earliest menarcheal age group. No evidence for an association between age at menarche and stroke or total cardiovascular death was found.

Although the association of menarche with risk of death from all causes is biologically plausible, the specific underlying mechanisms remain poorly understood. Early menarche has been consistently associated with adult obesity (6, 20). However, we found that the association between menarcheal age and death from all causes was still apparent in studies that adjusted for adult BMI, which indicates that mechanisms other than obesity may be involved. Low birth weight, rapid growth during infancy, and higher childhood BMI values are associated with earlier age at menarche (21, 22); therefore, history of early menarche could reflect earlier adverse metabolic imprinting. Menarche may be delayed in those with chronic conditions that can affect the risk of death, including malnutrition and anorexia nervosa (23, 24). This could explain the U-shaped association found in some studies. Early menarche is a risk factor for breast cancer morbidity (25) and death from all cancers (19), which indicates that cancer might account for part of the higher risk of death associated with early menarche. However, 2 cohort studies failed to show an association between menarcheal age and breast cancer death (26, 27).

Although we could not establish whether the observed difference in the effect of menarche on the risk of death from IHD between women who smoke and those who either do not smoke or who have a low prevalence of smoking reflects a causal interaction, our findings could provide insights into the underlying mechanism. Recently, smoking has been suggested to modify levels of reproductive hormones in women, including androgens, estrogens, 17-hydroxprogesterone, and sex hormone–binding globulin (28). Menarche is also associated with several hormonal changes which, when modified by smoking, might exert a different effect on cardiovascular outcomes. Smoking has also been associated with reduced body weight (29), which suggests that the increased risk of death from IHD in early-maturing smoking women might be mitigated by changes in adiposity.

Our results may have potential public health implications. The contribution of reproductive history, in particular menarche before the age of 12 years, for disease risk prediction should be considered in future studies. Furthermore, our findings suggest that data on early menarche, independent of BMI, may be a useful tool for future intervention strategies targeting modifiable factors shown to trigger menstrual onset, including early childhood growth (30), socioeconomic factors (31), and family environment (32).

This is the first meta-analysis of the association between age at menarche and death. It includes large cohorts from different countries. Available evidence was limited and quite novel because all included studies were published since 2007, and more than half were published after 2010.

Studies included in the review met most validity criteria. Potential for recall bias in exposure ascertainment was a major limitation in all studies; however, exposure misclassification is likely to be nondifferential and, thus, to bias the effect estimates toward the null. Additional errors might arise because of measurement of only baseline information (e.g., smoking) and reliance on self-reported measures. None of the studies included measures of childhood adiposity, a key determinant of menarche timing, nor did they include measures of other potential confounders, such as birth weight, infant growth, and nutrition. This lack of data meant that we were not able to assess the effect of early-life factors on the associations of menarche timing with mortality outcomes. Moreover, residual confounding from unmeasured socioeconomic or other factors associated with birth cohort cannot be excluded.

An effort was made to reduce confounding by using estimates reported from the most comprehensively adjusted models; however, this approach means that our results might be susceptible to overadjustment, a potential problem in studies that controlled for adult adiposity and chronic conditions (e.g., history of diabetes and hypertension), factors that could lie on the causal pathway between early menarche and mortality outcomes. However, presentation of data from the reviewed papers did not allow us to pool estimates that were unanimously adjusted for the same set of important confounders or to conduct meaningful sensitivity analyses to investigate the effect of potential mediators.

In contrast to the consistent inverse association between menarcheal age and the risk of death from all causes, evidence for the relationship between menarche and cardiovascular death outcomes was less robust. The interpretation of results is limited by a number of factors. First, discrepancies in the definitions of CVD deaths either between or within studies (e.g., changes in *International Classification of Diseases* codes from the ninth to the tenth revision) are a potential source of heterogeneity and misclassification. Second, meta-analysis was limited to comparisons between the lower and median menarcheal age groups, and it was not possible to quantify the full range of the association. Third, in some of the mortality outcomes (e.g., stroke), European countries were not represented, thus limiting the generalizability of the results.

Our review was also limited to death from all causes and death from cardiovascular causes, and it did not include death from specific noncardiovascular causes. Finally, although we performed a comprehensive literature search and tried to identify unpublished studies either through contact with authors or by searching in “grey literature” (e.g., conference proceedings), the potential for publication bias remains.

## CONCLUSIONS

The current meta-analysis provides evidence for a consistent inverse association between menarcheal age and the risk of death from all causes. Early menarche appeared to be particularly detrimental in terms of death from all causes, but no obvious beneficial effect was observed for later menarcheal ages. The effect of menarcheal age on the risk of death from all causes appeared to be independent of adult BMI, possibly indicating other direct mechanisms linking pubertal maturation to later health. Further cohort studies are needed to clarify the effects of early menarche on cardiovascular mortality outcomes and to confirm the possible modifying role of smoking.

## Supplementary Material

Web Material
