# Supplementary material for: Age at Menarche and Risks of All-Cause and Cardiovascular Death: A Systematic Review and Meta-Analysis
Source: Am J Epidemiol. 2014 Jun 11;180(1):29–40. doi: 10.1093/aje/kwu113 (PMC4070937; doi:10.1093/aje/kwu113)
Supplement: Web Material [file supp_180_1_29__index.html]

Age at Menarche and Risks of All-Cause and Cardiovascular Death: A Systematic Review and Meta-Analysis — Web Material 

# Age at Menarche and Risks of All-Cause and Cardiovascular Death: A Systematic Review and Meta-Analysis

## Web Material

Web Material

**Files in this Data Supplement:**

- Web Material - Docx file
