# Supplementary material for: Age at Menarche and Risks of All-Cause and Cardiovascular Death: A Systematic Review and Meta-Analysis
Source: Am J Epidemiol. 2014 Jun 11;180(1):29–40. doi: 10.1093/aje/kwu113 (PMC4070937; doi:10.1093/aje/kwu113)
Supplement: Web Material [file supp_kwu113_kwu113supp.docx]

| **Web Appendix 1**. List of Key Terms by Database and Limits Used in the Literature Search of the Current Review | | | |
| --- | --- | --- | --- |
| **Database** | | | |
| **MEDLINE (Pubmed)** | **EMBASE (Ovid)** | **Web of knowledge** | **Scopus** |
| 1. Menarche[Mesh] OR puberty[Mesh] OR "Puberty, Precocious"[Mesh] OR "Puberty, Delayed"[Mesh] OR menarche[All Fields] OR puberty[All Fields] OR menarch*[Title/Abstract] OR "pubertal onset"[Title/Abstract] OR "pubertal timing” [Title/Abstract] OR "sexual maturation"[Title/Abstract]) 2. Mortality[Mesh] OR mortality[Subheading] OR mortality[All Fields] OR death[Mesh] OR death*[All Fields] OR “cardiovascular mortality”[Title/Abstract] 3. epidemiological studies[MeSH Terms] OR cohort studies[MeSH Terms] OR cross sectional studies[MeSH Terms] OR case control studies[MeSH Terms] OR prospective studies[MeSH Terms] OR cohort[All Fields] OR longitudinal[All Fields] OR follow-up[All Fields] OR "observational study"[All Fields] OR "cross-sectional“[All Fields] OR "case-control"[All Fields] 4. #1 AND #2 AND #3 5. **#**4 Filters: Humans | 1. menarche.mp. or exp menarche/ 2. menarch*.ti,ab. 3. Puberty.mp. or precocious puberty/ or delayed puberty/ or exp puberty/ 4. exp Sexual Maturation/ 5. (precocious and puberty).ti,ab. 6. menstrua*.ti,ab. 7. (pubertal adj (onset or maturation or timing)).ti,ab. 8. 1 or 2 or 3 or 4 or 5 or 6 or 7 9. exp cardiovascular mortality/ or exp mortality/ or Mortality.mp. 10. mortality.ti,ab. 11. exp death/di, et [Diagnosis, Etiology] 12. 9 or 10 or 11 13. exp cohort analysis/ or cohort.mp. or exp follow up/ 14. prospective.mp. or exp prospective study/ 15. longitudinal.mp. or exp longitudinal study/ 16. exp retrospective study/ or retrospective.mp. 17. exp case control study/ 18. exp cross-sectional study/ 19. exp observational study/ 20. (cohort or longitudinal or follow-up or prospective).ti,ab. 21. 13 or 14 or 15 or 16 or 17 or 18 or 19 or 20 22. 8 and 12 and 21 23. limit 22 to human | 1. Title=(menarch*) OR Title=(pubert*) OR Topic=(menarche) 2. Topic=(mortality) OR Title=(mortality) OR Title=(death*) 3. Topic=(cohort OR longitudinal OR prospective OR case control OR follow-up OR cross-sectional OR observational OR retrospective) OR Title= (cohort OR longitudinal OR prospective OR case control OR follow-up OR cross-sectional) 4. #1 AND #2 AND #3 5. #4 Refined by: [excluding] Document Types=( REVIEW OR BOOK OR MEETING ) | 1. TITLE-ABS-KEY(menarch*) OR TITLE-ABS-KEY (puberty) 2. TITLE-ABS-KEY(mortality) OR TITLE-ABS-KEY(death*) 3. TITLE-ABS-KEY(cohort OR longitudinal OR prospective OR "case control" OR follow-up OR cross-sectional OR observational OR retrospective) OR ALL(cohort OR longitudinal OR prospective OR follow-up) 4. #1 AND #2 AND #3 5. #4 AND (EXCLUDE (DOCTYPE, "re") OR EXCLUDE(DOCTYPE, "ed") OR EXCLUDE (DOCTYPE, "sh") OR EXCLUDE(DOCTYPE, "le") OR EXCLUDE (DOCTYPE, "no") OR EXCLUDE(DOCTYPE, "bk")) |

# **Web Appendix 2: NEWCASTLE - OTTAWA Quality Assessment Scale**

COHORT STUDIES

*Note*: A study can be awarded a maximum of one star for each numbered item within the Selection and

Outcome categories. A maximum of two stars can be given for Comparability

Selection

1) Representativeness of the exposed cohort

A) truly representative of average postmenstrual women in the community *

B) somewhat representative of average postmenstrual women in the community *

C) selected group of users eg nurses, volunteers

D) no description of the derivation of the cohort

2) Selection of the non exposed cohort

A) drawn from the same community as the exposed cohort *

B) drawn from a different source

C) no description of the derivation of the non exposed cohort

3) Ascertainment of exposure

A) secure record (eg surgical records) *

B) structured interview *

C) written self report

D) no description

4) Demonstration that outcome of interest was not present at start of study (NOT APPLICABLE)

A) yes *

B) no

Comparability

1) Comparability of cohorts on the basis of the design or analysis

A) study controls for age*

B) study also controls for at least one indicator of socioeconomic status (eg. education, income) and at least one of the following lifestyle factors: physical activity, alcohol, diet

1) Assessment of outcome

A) independent blind assessment *

B) record linkage *

C) self report

D) no description

2) Was follow-up long enough for outcomes to occur

A) yes (≥10 years, or ≥7 years if mean age at baseline was >75) *

B) no

3) Adequacy of follow up of cohorts

A) complete follow up - all subjects accounted for *

B) subjects lost to follow up unlikely to introduce bias - small number lost - > 80 % follow up, or description provided of those lost *

C) follow up rate < 80% and no description of those lost

D) no statement

**Web Figure 1**. Combined funnel plot of the HR for the comparison between lower vs. median menarcheal age for the three cardiovascular mortality outcomes against their standard error. There is no obvious asymmetry or suggestion for “missing” small, negative studies.

| Web Table 1. Hazard Ratios and 95% Confidence Intervals Across Menarcheal Age Groups in Studies Looking on the Association Between Menarcheal Age and All-Cause Mortality | | | |
| --- | --- | --- | --- |
| First Author, Year (Reference No.) and MA Groups | HR | 95% CI | *p* value for linear trend |
| Jacobsen, 2007 ([1](#_ENREF_1)) |  |  |  |
| <13yrs | 1.09 | 1.05, 1.13 | <0.001 |
| 13yrs | 1.02 | 0.99, 1.05 |  |
| 14yrs | Referent |  |  |
| 15yrs | 0.97 | 0.94, 0.99 |  |
| 16yrs | 0.96 | 0.9, 1.0 |  |
| >16yrs | 0.97 | 0.93, 1.01 |  |
| Tamakoshi, 2011 ([2](#_ENREF_2)) |  |  |  |
| 9-12yrs | 1.16 | 1.01, 1.32 | 0.35 |
| 13yrs | 1.01 | 0.92, 1.11 |  |
| 14yrs | Referent |  |  |
| 15yrs | 0.97 | 0.9, 1.05 |  |
| 16yrs | 0.98 | 0.91, 1.05 |  |
| 17yrs | 0.92 | 0.84, 1.01 |  |
| 18-20yrs | 1.05 | 0.96, 1.14 |  |
| Lakshman, 2009 ([3](#_ENREF_3)) |  |  |  |
| 8-11yrs | Referent |  | 0.04 |
| 12yrs | 0.81 | 0.69, 0.95 |  |
| 13yrs | 0.86 | 0.75, 0.99 |  |
| 14 yrs | 0.87 | 0.75, 1.0 |  |
| 15-18yrs | 0.82 | 0.7, 0.95 |  |
| Jacobsen, 2009 ([4](#_ENREF_4)) |  |  |  |
| <11yrs | 1.45 | 1.18, 1.78 | <0.001 |
| 11yrs | 1.2 | 1.06, 1.36 |  |
| 12yrs | 1.11 | 1.01, 1.23 |  |
| 13yrs | Referent |  |  |
| 14yrs | 0.96 | 0.87, 1.06 |  |
| 15yrs | 0.96 | 0.85, 1.09 |  |
| 16yrs | 1.11 | 0.96, 1.3 |  |
| >16yrs | 0.88 | 0.69, 1.13 |  |
| HR=hazard ratio, CI=confidence interval, MA: menarcheal age | | | |

| Web Table 2. Hazard Ratios and 95% Confidence Intervals Across Menarcheal Age Groups in Studies Looking on the Association Between Menarche and Cardiovascular Mortality Outcomes | | | | | | | |
| --- | --- | --- | --- | --- | --- | --- | --- |
| First Author, Year  (Reference No.) | MA category | IHD | | Stroke | | CVD | |
|  |  | HR | 95% CI | HR | 95% CI | HR | 95% CI |
| Gallagher, 2011 ([5](#_ENREF_5)) | ≤13yrs | 1.44 | 1, 2.05 | IS: 1.05 | 0.75, 1.45 |  |  |
|  |  |  |  | HS:0.97 | 0.79, 1.19 |  |  |
|  | 14yrs | 1.06 | 0.76, 1.47 | IS: 0.82 | 0.61, 1.09 |  |  |
|  |  |  |  | HS: 1.03 | 0.88, 1.22 |  |  |
|  | 15yrs | Referent |  |  |  |  |  |
|  | 16yrs | 1.09 | 0.82, 1.45 | IS: 0.97 | 0.76, 1.23 |  |  |
|  |  |  |  | HS: 0.92 | 0.79, 1.07 |  |  |
|  | ≥17yrs | 0.85 | 0.65, 1.12 | IS: 0.87 | 0.7, 1.09 |  |  |
|  |  |  |  | HS: 0.9 | 0.79, 1.03 |  |  |
| Cui, 2006 ([6](#_ENREF_6)) | ≤13yrs | Referent |  |  |  |  |  |
|  | 14yrs | 0.77 | 0.41, 1.45 | 1.29 | 0.89, 1.88 | 1.13 | 0.88, 1.45 |
|  | 15yrs | 1.22 | 0.7, 2.11 | 1.03 | 0.71, 1.49 | 1.06 | 0.83, 1.35 |
|  | 16yrs | 0.98 | 0.55, 1.73 | 1.42 | 1, 2.02 | 1.13 | 0.89, 1.44 |
|  | ≥17yrs | 1.28 | 0.75, 2.2 | 1.32 | 0.93, 1.87 | 1.22 | 0.96, 1.53 |
| Lakshman, 2009 ([3](#_ENREF_3)) | 8-11yrs |  |  |  |  | Referent |  |
|  | 12yrs |  |  |  |  | 0.83 | 0.63, 1.09 |
|  | 13yrs |  |  |  |  | 0.75 | 0.58, 0.97 |
|  | 14 yrs |  |  |  |  | 0.93 | 0.73, 1.18 |
|  | 15-18yrs |  |  |  |  | 0.79 | 0.61, 1.03 |
| Jacobsen, 2009 ([4](#_ENREF_4)) | <12yrs | 1.37 | 1.09, 1.73 | 1.43 | 1.02, 2.01 |  |  |
|  | 12yrs | 1.2 | 0.98, 1.48 | 1.17 | 0.86, 1.59 |  |  |
|  | 13yrs | Referent |  |  |  |  |  |
|  | 14yrs | 0.93 | 0.76, 1.14 | 1.14 | 0.86, 1.52 |  |  |
|  | 15yrs | 0.95 | 0.74, 1.23 | 0.78 | 0.52, 1.17 |  |  |
|  | >15yrs | 1.14 | 0.88, 1.49 | 0.9 | 0.59, 1.37 |  |  |
| Mueller, 2012 ([7](#_ENREF_7)) | Ever smokers | | | | | | |
|  | ≤12yrs | 0.75 | 0.42,1.33 | 0.68 | 0.27, 1.75 | 0.65 | 0.41, 1.03 |
|  | 13-14yrs | Referent |  |  |  |  |  |
|  | 15-16yrs | 0.77 | 0.55,1.08 | 0.66 | 0.4, 1.08 | 0.71 | 0.55, 0.91 |
|  | ≥17yrs | 0.78 | 0.51,1.19 | 1.26 | 0.74, 2.17 | 0.9 | 0.67,1.22 |
|  | Never smokers | | | | | | |
|  | ≤12yrs | 1.01 | 0.78, 1.3 | 1.2 | 0.84, 1.7 | 1.02 | 0.82, 1.24 |
|  | 13-14yrs | Referent |  |  |  |  |  |
|  | 15-16yrs | 0.82 | 0.7, 0.97 | 1.13 | 0.91, 1.4 | 0.95 | 0.85,1.07 |
|  | ≥17yrs | 0.79 | 0.64, 0.97 | 0.95 | 0.72, 1.26 | 0.86 | 0.74,1.01 |
| Chang, 2011 ([8](#_ENREF_8)) | 10-16yrs | Referent |  |  |  |  |  |
|  | 17-18yrs | 0.49 | 0.25, 0.98 | 0.98 | 0.74, 1.29 | 0.94 | 0.75, 1.18 |
|  | >19yrs | 0.65 | 0.31, 1.35 | 0.91 | 0.66, 1.25 | 0.94 | 0.73, 1.21 |
| HR=hazard ratio, CI=confidence interval, MA: menarcheal age, IS: Ischemic Stroke, HS: Haemorrhagic Stroke, IHD: Ischemic Heart Disease, CVD: Total Cardiovascular Disease | | | | | | | |

References

1. Jacobsen BK, Heuch I, KvΓ¥le G. Association of low age at menarche with increased all-cause mortality: A 37-year follow-up of 61,319 Norwegian women. *Am J Epidemiol* 2007;166(12):1431-1437.

2. Tamakoshi K, Yatsuya H, Tamakoshi A. Early age at menarche associated with increased all-cause mortality. *European journal of epidemiology* 2011;26(10):771-778.

3. Lakshman R, Forouhi NG, Sharp SJ, et al. Early age at menarche associated with cardiovascular disease and mortality. *The Journal of clinical endocrinology and metabolism* 2009;94(12):4953-4960.

4. Jacobsen BK, Oda K, Knutsen SF, et al. Age at menarche, total mortality and mortality from ischaemic heart disease and stroke: the Adventist Health Study, 1976-88. *International journal of epidemiology* 2009;38(1):245-252.

5. Gallagher LG, Davis LB, Ray RM, et al. Reproductive history and mortality from cardiovascular disease among women textile workers in Shanghai, China. *International journal of epidemiology* 2011;40(6):1510-1518.

6. Cui R, Iso H, Toyoshima H, et al. Relationships of age at menarche and menopause, and reproductive year with mortality from cardiovascular disease in Japanese postmenopausal women: the JACC study. *Journal of epidemiology / Japan Epidemiological Association* 2006;16(5):177-184.

7. Mueller NT, Odegaard AO, Gross MD, et al. Age at menarche and cardiovascular disease mortality in Singaporean Chinese women: the Singapore Chinese Health Study. *Annals of epidemiology* 2012;22(10):717-722.

8. Chang HS, Odongua N, Ohrr H, et al. Reproductive risk factors for cardiovascular disease mortality among postmenopausal women in Korea: the Kangwha Cohort Study, 1985-2005. *Menopause* 2011;18(11):1205-1212.
